# Smart and Climate-Smart Agricultural Trends as Core Aspects of Smart Village Functions

**DOI:** 10.3390/s20215977

**Published:** 2020-10-22

**Authors:** Adegbite Adesipo, Oluwaseun Fadeyi, Kamil Kuca, Ondrej Krejcar, Petra Maresova, Ali Selamat, Mayowa Adenola

**Affiliations:** 1 Department of Soil Protection and Recultivation, Brandenburg University of Technology, Konrad-Wachsmann-Alle 6, 03046 Cottbus, Germany; adesiade@b-tu.de; 2 Department of Geology, Faculty of Geography and Geoscience, University of Trier, Universitätsring 15, 54296 Trier, Germany; phar2kind@gmail.com; 3 Center for Basic and Applied Research, Faculty of Informatics and Management, University of Hradec Kralove, Rokitanskeho 62, 50003 Hradec Kralove, Czech Republic; kamil.kuca@uhk.cz (K.K.); aselamat@utm.my (A.S.); 4 Malaysia Japan International Institute of Technology (MJIIT), Universiti Teknologi Malaysia, Jalan Sultan Yahya Petra, Kuala Lumpur 54100, Malaysia; 5 Department of Economy, Faculty of Informatics and Management, University of Hradec Kralove, Rokitanskeho 62, 500 03 Hradec Kralove, Czech Republic; petra.maresova@uhk.cz; 6 Department of Urban and Regional Planning, School of Environmental Technology, Federal University of Technology, PMB 704, Akure 340252, Nigeria; adenolamt@gmail.com

**Keywords:** smart village, smart agriculture, climate-smart agriculture, technology, sustainability

## Abstract

Attention has shifted to the development of villages in Europe and other parts of the world with the goal of combating rural–urban migration, and moving toward self-sufficiency in rural areas. This situation has birthed the smart village idea. Smart village initiatives such as those of the European Union is motivating global efforts aimed at improving the live and livelihood of rural dwellers. These initiatives are focused on improving agricultural productivity, among other things, since most of the food we eat are grown in rural areas around the world. Nevertheless, a major challenge faced by proponents of the smart village concept is how to provide a framework for the development of the term, so that this development is tailored towards sustainability. The current work examines the level of progress of climate smart agriculture, and tries to borrow from its ideals, to develop a framework for smart village development. Given the advances in technology, agricultural development that encompasses reduction of farming losses, optimization of agricultural processes for increased yield, as well as prevention, monitoring, and early detection of plant and animal diseases, has now embraced varieties of smart sensor technologies. The implication is that the studies and results generated around the concept of climate smart agriculture can be adopted in planning of villages, and transforming them into smart villages. Hence, we argue that for effective development of the smart village framework, smart agricultural techniques must be prioritized, viz-a-viz other developmental practicalities.

## 1. Introduction

The need to develop rural communities in terms of productivity and convenience, so as to curb urban migration has received much attention in the last decade. First, the Institute of Electrical and Electronics Engineers (IEEE), as part of its mission, commenced the installation of solar-powered bulbs in many rural communities worldwide [1]. This was followed in 2016 by the Cork Declaration, agreed amongst 340 representatives of European states towards ensuring that rural communities enjoy better lives. These efforts culminated into the coining of the word “smart village”, defined as a community that tries to develop current strength and resources, while making futuristic developmental plans on the basis of technology [2,3]. While there are several thematic areas of priority within the smart village development framework, agriculture is seen as the most important of them all [3]. Furthermore, the need to bridge the digitization gap between cities and villages, is also an important aspect, so that lives and livelihood can be improved. Since a smart village is one that seemingly accepts new technologies, precision agriculture uses ultra-modern techniques for animal and crop production, which saves time and reduces wastage, and meets the requirements of smart villages. This is crucial for the sustainability of smart villages [4]. This is because improved food production and efficient animal management systems must be at par with village development, and must be continually transformed to influence the different aspects of smart villages, in terms of policy and practice [5]. 

To effectively play its role in smart villages, precision agriculture covers smart and climate smart agriculture (CSA) techniques, and other aspects that are capable of ensuring higher agricultural production output in an environment-friendly manner, provides optimum income for the farmer, and is able to feed a growing population. Many studies showed that these processes can be realized through the adoption of ultra-modern agricultural techniques such as bio and nano technologies [6], IoT and blockchain-based methods [7], and drone technologies [8], among other climate smart ideas. On the basis of this argument, efforts that tend to reduce farming losses, increase yield, as well as monitor, detect, and potentially prevent plant and animal diseases are now being automated, finding growing applications, and offering optimal solutions. Based on the forgone explanations, the current study attempts to establish smart and CSA trends in smart village research, in order to see how much they are useful for smart village development. 

The rest of this study is arranged as follows. Section 1.1 draws a foundation for this study, by focusing on the research question. Section 2 briefly builds a background for smart village research by listing existing projects, and describes a few state-of-the-art smart agricultural solutions. In Section 3, attention is drawn to climate-smart agriculture, with specific reference to what makes up the concept, a few challenges in its framework, as well as the latest progress in its development. Section 4 describes the challenges created by the interplay of adopting CSA in smart villages, and also tries to answer the research question. The section also conceptualizes climate-smartness, as it influences sustainable development of smart villages. Finally, Section 5 describes future research directions in smart-village and smart agricultural research, and draws relevant policy recommendations and conclusions

### 1.1. Research Question

Based on the vast importance of agriculture in smart village development, this study adapts its research question from the editorial note presented by the editors of MDPI’s special issue within the Sustainability journal published in August 2018. Within the report, Visvizi and Lytras [9] gave a revealing background of future directions for smart village research. The editors pointed to a few research questions that future smart village research should strive to answer. One of these is: “How will smart and CSA research give account of, and conceptualize transformation and change in the smart village context?”(p. 8) [9]. This question is what the current study modifies and seeks to answer. 

## 2. Related Literature

### 2.1. Current Smart-Village Projects Around the World

Before delving completely into smart agricultural systems in smart villages, it is important to consider existing smart village initiatives in order to have an updated knowledge of smart village trends, and why smart agriculture might need to be prioritized. Zavratnik et al. [10] described the IEEE smart village project, and the EU smart village initiative, which are further elaborated in subsequent paragraphs. 

The IEEE smart village program is one of the most popular today. It has a goal of advancing education in off-grid societies, and fostering sustainability in the entire value chain of the smart village energy sector. Initially taking off as an initiative that seeks to provide community solutions in 2009, the current name was coined 5 years later. The IEEE smart village plan is a global initiative, touching lives in Asia, some parts of North America, and mostly in Africa [1], through the promotion of smart energy production in rural areas, and is mostly financed through fundraising. Major efforts that were developed from the initiative include the so-called SunBlazer II—a movable power base solar station [11]; “Learning beyond the Light Bulb” [12]—a program aimed at training locals on the development and design of off-grid solar electricity panels and fostering its sustainability and scalability. As reported by Zavratnik [10], the program also comes with a remote study event that runs for about nine months, and allows practice exchange amongst involved communities, for knowledge sharing and skill enhancement. 

Within the framework of the Consultative Group on International Agricultural Research (CGIAR), several smart-village projects took off around the world. Many of which were funded through international research organizations with clear impacts in areas that were worse hit by climate change [10]. These projects mostly focus on training smallholder farmers on agricultural resilience, through the adoption of practices that support food security [13], so that persons within these affected communities are able to maintain a livelihood through agricultural methods that help decrease GHG emissions. For instance, farmers in the Lower Nyando valleys of Kenya are benefitting from improved agroforestry systems that adopts knowledge of Information and Communication Technology (ICT) [13]. Thanks to the CGAIR initiative, they are able to cultivate cash crops in-between rows of multi-purpose trees, thereby improving soil stability and enrichment. Given the increased demand for trees, several nurseries were developed, adding farmers’ incomes, and with women as the highest beneficiaries. The state of Bihar in India also benefitted from the CGIAR’s smart village initiative. It previously had soils that were greatly affected by water-logging, but new drainage construction changed the channel of rapidly flowing flood waters, out of the farming areas [14]. This improved system also ensured that underground aquifers were steadily recharged. Improved technological ideas also saw better rainwater harvesting in areas benefitting from the Climate Change Agriculture and Food Security (CCAFS) program. Overall, weather and planting can now be monitored from smartphone applications by the farmers, in order to avoid unwanted losses [15].

The European Union’s smart village initiative is by far the most organized and detailed system. Having undergone several fine-tuning, the initiative has improved tremendously since the Cork Declaration 2016 [10]. Notable amongst the goals of the EU smart village drive is agricultural boost, mainly because the rural areas are where European foods are mostly produced [16]. There is also the goal of reduced youth exodus to urban centers [17]. In the first assembly of the newly adopted “Intergroup SMART Villages for Rural Communities”, György Mudri, a former Members of European Parliament stressed that smart villages are not only for the development of new infrastructures, but also for building capacity of locals [18]. In response to this statement, The Austrian Chamber of Agriculture commenced online training for about 10,000 farmers, who now have remote access to latest agricultural researches and can subsequently implement such ideas on their farms [19]. There is also the so-called COWOCAT rural initiative, which currently trains youth to commence working in villages [19].

Description of smart village drives of the above initiatives show that agriculture is one of the most prominent aspects of the smart village plans. As a result, this study delves into smart agricultural practices that can the build capacity of smart villages, if adopted.

### 2.2. Ultra-Modern Smart Agricultural Solutions

While there are varieties of smart technologies adopted in agriculture nowadays, this section focuses only on bio-sensors, agricultural drones, IoT and Blockchain-based sensors, and a number of combined technologies that adopted animal husbandry, as well as in crop, soil, and pest management. 

### 2.3. Nanostructured Biological Sensors 

Biological sensing devices are some of the new technological interventions reshaping agricultural systems today, which might be adopted in smart villages. As reported by Antonacci [6], bio-sensors with extremely small structures were found to possess the ability to help in crop maturity evaluation, management of amount of pesticides and fertilizers, as well as detection of humidity levels in soils for effective irrigation. To carry out these functions, bio-sensors rely on the characteristics of nano-materials, such as immobilizing bio-receptors on transducers, integrating and miniaturizing some biological components of plants, transducer systems, and micro-fluids, into very complex plants make-up [20,21]. Although the use of harmful pesticides is gradually being phased out in agricultural systems, less harmful pesticides are still very much in use [22]. In areas known for prior pesticides usage, modern agricultural techniques often aim at detecting pesticide presence, as well as their levels within the soil, before cultivation. To do this, cutting-edge bio-sensors with very high sensitivity (because of their surface-volume ratio), extremely rapid response time, and quick electron-transfer kinetic are utilized. The sensors possess stable strength to map pesticide quantities within soils, and longer lifespan, when compared to the earliest bio-based sensors [23]. Newly improved bio-sensors with extremely small structures are also able to surpass soil pre-treatment, due to the presence of pesticides, herbicides, and fungicides, without losing their potency [6].

Yu et al. [24] developed tyrosinase/TiO2 biosensor to determine the presence of atrazine pesticides. This was done by fabricating a structure through the allowance of vertical growth of TiO_2_ nanotubes. This meant that well-arranged nanotubes would provide large surface areas for immobilizing the tyrosinase enzyme. The structure gave room for excellent loading of enzymes, as well as transfer of electrons, which yielded improved system robustness and sensitivity. The system was tested in well-grinded, air-dried paddy soils, gathered at varying depths. The soil also passed through a sieving process using a 1.0 mm filter, and a 35 °C re-drying process that lasted for 48 hrs. It was subsequently mixed with acetone, prior to undergoing shaking at a temperature of 25 °C for 60 min. Results given by [24] showed that after carrying out analysis of supernatants, atrazine was observed to be present in 0.2 ppt to 2 part-per-billion. Standard deviation was subsequently found to be below 0.05 ppt when compared to high performance liquid chromatography (HPLC).

Dong et al. [25] introduced a novel nano-structured bio-sensor technique for detecting very low pesticide traces in soil. The technique works by electrochemically reducing Ellman’s reagent via the inhibition of acetylcholinesterase. This bio-sensor adopts amperometric, designed to immobilize acetylcholinesterase on multiple walls of carbon-type nanotubes-chitosan nanocomposites modified glassy carbon electrode. High sensitivity of the system is offered by the very good conductivity and biological compatibility of multiple walls of carbon-type nanotubes-chitosan [25]. This can be additionally improved by electrochemically reducing 5,5-dithiobis (2-nitrobenzoic) acid. In testing the system, methyl parathion pesticide was observed to exhibit an inhibitive effect on acetylcholinesterase. An electrochemical change in the reduction response of 5,5-dithiobis (2-nitrobenzoic) acid was also observed. Overall, the system was found to possess a pesticide detection precision of 7.5 × 10^−13^ M when tested on spiked soil. 

In another nano structured bio-sensor study, Shi et al. [26] observed the presence of soil acetamiprid using SELEX; a new 20 mer bio-sensing unit that is able to bind acetamiprid, using aptamer made of nanoparticles of gold. The unit works to detect the pesticide optically at values ranging from 75 nM to 7.5 µM. It bears the combined characteristics of a nanomaterial, and those of artificial molecules. Tested soils were collected around Tongji University, China, with initial air-drying carried out before the sample was grinded, to allow 1.0 mm sieving. A second drying was also done using an oven at 35 °C for 2 days, prior to acetone mixing and shaking at 25 °C for 60 min. Dichloromethane was subsequently added to the mixture, and then removed ultrasonically before the sample was filtered. 

Beyond sensing pesticides within soils, bio-sensors with very tiny structures were also employed in monitoring diseases of crop plants. A very important aspect of any smart village is the effective management of farm economy, achievable through the protection of crops against diseases. Quantum dots offer classical examples of materials that are useful for monitoring plant diseases, as they possess broad excitation spectra. Safarpour et al. [27] identified the vector responsible for sugar beet’s yellow vein and Rhizomania disease like Polymyxa betae. This was detected using quantum dot techniques that subjected the plant root sap samples to several pre-treatment in order to extract the virus. The quantum dots unit utilizes Förster Resonance Energy Transfer (FRET) modeling in its detection operation [27]. By using a similar technology, Bakhori et al. [28] detected synthetic oligonucleotide of Ganoderma boninense. However, this work employed adjusted quantum dots with carboxylic groups that are then conjugated using a DNA probe. This gave rise to an improved sensitivity of the system, yielding 3.55 × 10^−9^ M as the detection limit [28].

By adopting bio-sensors in the detection of soil nutrients and fertilizers, Ali et al. [29] revealed that soil nitrates can be detected using a system that relies on microfluidic impedimetric sensing. The unit works by adopting nano-sheets of graphene oxide and the nanofibers of the so-called poly (3,4-ethylenedioxythiophene). The researchers showed that poly (3,4-ethylenedioxythiophene) composite can bear the enzyme; nitrate reductase, and also measure the amount of nitrate ions in soil samples on which sweet corn was cultivated. This is done at 0.44-442 mg/L concentration, so the detection limit was 0.135 mg/L [29]. Carrying out the procedure, however, involves sample drying at 105 °C, and subsequent nitrate extraction through the addition of 2 M KCl solution. The mixture was shaken for 60 min, and filtered using Whatman filter paper. Finally, sample extraction was kept in a syringe for infusion into the experimental device.

Several other research examples exist for bio-sensor utilization in agricultural work. Nevertheless, a summary of some state-of-the art techniques, some of which are already described elsewhere, is presented in Table 1.

### 2.4. Drone Technologies (Unmanned Aerial Vehicles)

Unmanned Aerial Vehicles (UAV), also known as drones, have become popular in agricultural production work. In a review study by Mogili [8], the researchers reported that drones can be used in pesticide and fertilizer application, so that humans do not come in contact with the some of these pesticides, which are harmful, and are gradually being phased out. Drones can also function as water sprinkling systems [8,36]. 

Primicerio et al. [37] adopted VIPtero, a UAV for managing a vineyard in an experimental set-up in Italy. The system, which is made up of an aerial platform with six rotors and a camera, can fly in a self-governed manner to a particular point in the air, in order to take measurement of the vegetation canopy reflectance. Prior to flight take-off, accuracy of the camera is evaluated in relation to ground-based measurements with high resolution, which were gathered using field spectrometer. Subsequently, VIPtero gets air-bound in the vineyard, and gathers as many as 63 multi-spectral images in a 600 seconds time period. The recorded images are analyzed and classified, prior to the production of vigor maps on normalized difference vegetation index. Results showed the heterogeneity conditions of the crops, implying that they were in line with those gathered using the ground-based spectrometer [37]. This smart system appears to be promising as an effective and detailed data gathering system in agriculture, and can be adopted over larger areas in smart villages.

In another UAV based research, Burgos et al. [38] used a 4 cm Sensefly Swinglet UAV to differentiate green cover from grape canopy. A digital surface model (DSM) with 3 dimensions was adopted to create an exact digital terrain models (DTM), acquired via the use of processing libraries of python, and subsequently subtracted from DSM, so as to arrive at a differential digital model (DDM) for the measured terrain (a vineyard). Vine pixels within the DDM were obtained by selection of pixels >50 cm elevation from the ground. The results indicated that there is a possibility of separating vine row pixels from green cover pixels, as a differential digital model pointed to values ranging from –0.1 m to +1.5 m. Furthermore, manual polygon delineation, which depended on an RGB image of the vine rows and green cover, revealed huge differences averaging 1.23 m and 0.08 m for vine and ground, respectively. Elevation of the vine rows was good and tallied with its topping height of 1.35 m from the field [38]. The authors noted that vine pixels extraction would aid future analyses, such as pixels’ supervised classification.

Berni et al. [39] also demonstrated the possibility of generating remotely sensed data over an agricultural field, using a UAV that had a relatively cheap narrowband and thermal multispectral imaging sensors of 20 cm and 40 cm resolutions, respectively. The system gave rise to surface reflectance and temperature data, after adapting MODTRAN-based atmospheric correction. Biophysical parameter estimation was carried out using a number of vegetation indices, leading to the production and validation of chlorophyll content, detection of water stress from PRI index, as well as the temperature of the canopy. These results showed that the system yielded the same results as the conventional, expensive, and risky manned airborne sensors. 

### 2.5. IoT-based Sensors with Complimentary Blockchain Technology

Many villages face severe agricultural challenges and that require upgrading to smart agriculture, which offers a wide range of state-of-the-art solutions. For example, in villages where access to water is a challenge, Khoa [7] maintained that IoT-based sensors can be useful in water-management irrigation systems on large rural farms. In their research, the authors developed a novel system that is able to monitor soil water level and schedule sprinkling/spraying times in well-calculated amounts. This relatively cheap technique, functions by receiving real-time data from sensors fixed within strategically arranged tunnels, in and around the farm. Based on the information supplied by sensors, which can be received through a mobile phone application, the user might decide to water the farm. Subsequently, when soil water level increases to an optimal level, the system notifies the user, who can remotely or manually switch-off the water-pumps. A unique feature of this system is its usability in up to two farms [7]. In a similar study, Nagpure et al. [40] described another IoT-based system that works by using a similar routine as [7]. However, two differences include; scaring animals away using current pulses, and wireless sensor monitoring of the ecological conditions (e.g., altitude and humidity) to ascertain the amount of irrigation water needed each time [40], which the latter unit possesses. 

Mat et al. [41] presented an IoT-based mushroom cultivation, which produced a better yield when compared to the conventional system. This tool is based on an automated sensors for fertilizer application and water sprinkling on the farm, which can be controlled from the farmer’s mobile phone or manually, from a centralized point within the farm. The system ensures that the timing for wetting the crop is strictly adhered to, so that the farming operation can progress even without the farmer. Overall, it was observed that average mushroom size in thickness and weight exceeded conventional cultivation by 0.3 cm and 5 gram, respectively.

As reported by Prathibha [42], it is important to curb the effect of environmental conditions on crop yield output. To do this, an efficient measurement method of the elements of weather might be required. Prathibha’s research, therefore, proposed a CC3200 combined sensor unit, which comprises a processor for network, a micro-controller, a Wi-Fi unit, a camera, as well as temperature and humidity sensors. This weather utility device comes as a portable unit with low power consumption for longer battery-life. The system monitors temperature (using a thermopile sensor that uses infrared technology) and humidity across the agricultural field, which are subsequently processed as camera images and sent via Wi-Fi to the farmer’s mobile phone as multi-media messages. Information of this nature helps the farmer to know how good the soil water is to support the grown crop. A similar study [43] designed another unit that can also send immediate signals to a farmer, after recording real-time data on weather, in and around the farm. This unit was made up of a breadboard, a combination of sensors that can monitor UV Index/IR/Visible light (SI1145 Digital Sensor), soil moisture content, humidity, temperature (DHT11), an ESP32s Node MCU, all of which are connected to a monitor, which is in turn linked remotely to the famer’s mobile phone, using an LED visual alert and Blynk mobile phone application. Two very special characteristics of this unit are; its ability to save power in sleep mode, for a battery life that averages 10 days, and the speed of sending signal (180 s) [43]. 

By using a pre-coded algorithm, also known as the “*Cuckoo Search Algorithm*” [44], a framework for automated watering of a piece of farmland was designed. Based on pre-analysis of different kinds of soils, the researchers found that a soil moisture value of 700 meant dry, and would require immediate watering. The IoT sensor was, therefore, designed on the basis of this information, comprising the so-called ThingSpeak, which also gives direction on the most suitable soil type for a specific crop. A temperature-based sensor was initially used on the soil, and the result was sent to a converter called Arduino. Depending on the measured value, the Arduino was connected to an automated watering system, which could be controlled from the mobile device handled by the farmer. When soil wetting gets to optimal levels, the soil sensor sends a feedback signal to the farmer who then stops the watering [44].

While IoT-based sensing techniques are available for improving precision agriculture, optimizing these ideas with blockchain technologies might offer even more robust results. Patil et al. [45] noted that IoT-based sensing technologies might sometimes be flawed on the grounds of; extremely large scale, a lack of homogeneity of different IoT-based sensing operations, as well as standardization. This meant that the data gathered using IoT-based sensing technologies comes with privacy concerns for the farmer [45]. Hence, the researchers developed a blockchain greenhouse farming tool to cater to security and privacy. The model is made up of a smart greenhouse (a covered piece of farmland protected from environmental conditions) that comprises a series of sensors and actuators, smart hub (a local blockchain which manages the connectivity of all sensors and equipment in the smart greenhouse); an overlay network connection that manages the nodes; a cloud storage platform and an end-user platform. A system of this nature addresses security challenges across all fronts within the farm [45]. In another Blockchain-based smart agricultural study [46], a traceability platform for food safety was designed. In collaboration with the Internet of Things, the system involved “Enterprise Resource Planning” where farmers, processing plants, and organizations involved in the logistics of agricultural and food products, and the consumers, can assess on their mobile phones, a blockchain node that gives detailed description of how the products were cultivated, harvested, stored, processed, and sold [46]. The essence of this technique was to build virtual trust in food processing, using the so-called *Trusted Trade Blockchain Network Cloud Platform (TTBNCP)*

### 2.6. Smart Animal Production, Management, and Monitoring

The use of machine vision in body condition scoring of dairy received extensive research attention in the last few decades. Fox et al. [47] listed animal nutrition, insemination, and health as core reasons for body condition monitoring. When this process is carried out by the farmer or veterinary doctor (human monitoring), there is a possibility for biased data gathering, due to the individual’s mental state, level of experience, and residual knowledge [48]. Furthermore, the process might also be time-consuming. Improvement in body condition scoring in the 1970s employed ultrasounds [49], which was flawed on the ground that mastering the collection of reliable ultrasonic body condition scoring required more time in comparison to data gathering by humans [50]. Additionally, the cost of purchasing the ultrasound device, and hiring an expert, made the process too expensive. This led to camera-recording of animals applied to body condition monitoring, based on the belief that this would yield better results [48]. Fourier descriptor cameras [51], thermal [52], and RGB cameras [53] were adopted. Nevertheless, images could not be processed automatically until seven years ago [54]. As a step in the right direction for body condition scoring, Spoliansky et al. [55] used a 3D camera that was well-equipped to carry out automatic image processing, leading to the development of effective and unbiased collection of body condition scoring, in 2017 [55]. The system provides real-time data, useful for commercial milking purposes, and genetic evaluation (based on lactation). In addition, automated image gathering of body condition scoring might provide ease of monitoring when there are more than one animal. This is pivotal to early warning signs for morphological changes in animal body size [48].

Yanmaz et al. [56] suggested adopting thermography in the early detection of lameness in horses. Two types of thermography are—contact and contactless types. Contactless thermography has higher precisions via infrared radiation. Internal temperature of the affected animal part can be viewed on a medical thermogram, so that treatment can be planned early. Similarly, temperature around a sick cattle’s gluteal region differs significantly from those of other parts when studied using thermal infrared scanning [57]. As reported by Steensels et al. [58,59], temperature management in poultry, as well as early mastitis identification are some other areas where thermal scanning was reported to be useful.

Accelerometers exist nowadays for remote measurement of animal gait [60]. This can determine when the animal is lying or walking [48]. The method is also useful in the determination of lameness, animal sickness, or how much the animal feeds [61], based on the distance covered by the animal against time. The device can be mounted on the leg, ear or other parts of the animal, so that it continuously sends signals to the farmers’ mobile phone. When the animal is lying down, the instrument automatically changes its processing speed. The device was also utilized in monitoring the health of fishes, by attaching it to their fins [62]. A demerit of the system, however, is the fact that continuous processing of mobility data tends to rapidly reduce battery life [63].

Wearable belts that are able to tap animal sweat and measure the amount of sodium it contains are some of the latest smart technologies in animal husbandry. In the work of Glennon et al. [64], the authors developed a smart technique for quick detection of the sodium content of sweat. The unit appears in two ways, one that resembles a vertically placed watch and can be worn round an animal leg, and the other that looks like a horizontal pod. Both come with a Velcro strap that can be used to attach it to the animal skin. The systems, through capillary action, receive sweat via its orifice, and send it through an electrode that is sensitive to sodium. The electrode in turn sends it into a storage section containing an adsorbent substance. Sweat flow rate can be improved by varying the width of the sweat flow channel in-between electrode and the storage section of the system. Sweat flow rate generally decreases with decreased width of the flow channel. This also determines the length of time the system will be used before the electrode and the adsorbent material are changed. Stored sweat is available for measurement as total harvested sweat volume as well as its sodium concentration per time. Electrode signals are moved to an electronic board that possesses high input impedance for voltage capturing. Results of this analysis are sent to a remote base station, which is either a laptop, or mobile phone, using bluetooth technology for onward visualization and possible storage. 

In livestock management [65] a pregnancy detection method was developed, which makes use of Xbee transmitters linked to LM35 temperature sensors. Two animals were experimented, with temperatures recorded at five days and twelve days from insemination. The sensing system was attached to the tail of the cows. Temperature records were found to be high in pregnant cow, especially in the evenings. The sensing unit works effectively within a distance of 40 m, and serves as a low-cost technique, when compared to some invasive pregnancy detection methods in livestock.

In an ongoing study aimed at finding a sensor that is able to detect the level of progesterone in milk fed to cattle [66], interdigital sensors are used, which are able to yield single side access to the substance being tested. Within the study, progesterone hormone of about 20 mg was allowed to dissolve in 0.5 mL of approximately 100 per cent ethanol. The solution was subsequently poured into about 1000 mL of pure Milli-Q water, so that a stock solution was achieved. Successive mixture dilution led to 0.02 ng/mL progesterone concentration. The sensor offered different results at varying progesterone concentration with sensitivity in the pitch range of 50 µm [66].

Infectious coughs in piggery need rapid detection and treatment. To detect this kind of cough, Ferrari et al. [67] fixed 1 m multi-directional microphones of 50 to 16,000 Hz around a farm. The microphone was connected to a laptop, and animal cough sound patterns were recorded, digitized, and analyzed using Matlab 7. Having earlier injected healthy animals with citric acid, acoustic parameters such as time difference between coughs, peak frequency, and root mean square were used to differentiate coughs from a healthy pig from those of infected ones. While healthy pigs relaxed for about 52 s after each cough attack, the infected pigs coughed after every 37 s. Peak frequency for infected and non-infected pigs was observed to be 1600 Hz and 600 Hz, respectively.

## 3. Moving towards Climate-Smart Agriculture

Having established in Section 2.1 that agriculture is one of the most important factors to be considered in smart village development, it is crucial to stress that climate change is a major stressor for agricultural development of rural communities [68]. The implication is that developing an agriculturally-smart village entails accepting the concept of climate-smart agriculture. Agricultural risk posed by climate is a threat to food security. As a result, there is an urgent need to effectively manage agricultural production, while fighting climate change through adaptation, resilience and mitigation [69]. This is what climate-smart agriculture offers.

There is currently no unified definition for climate-smart agriculture (CSA). In fact, almost every new study within the framework of smart-agriculture, views CSA in a slightly unique way. Nevertheless, to build a strong foundation for climate-smart agricultural framework in smart village development, the current study adopts existing knowledge and definitions, to coin a new and more robust definition for the term. Table 2 presents some definitions put forward by climate change and agricultural scholars and research organizations. Keywords derived from the definitions show that each has one or more shortcomings. As a result, it might be difficult to build the concept of smart village on a definition that lacks one or more fundamental aspects.

Given the definitions in Table 2, considerable aspects of climate-smart agriculture include; capacity building, sustainability, emission reduction, vulnerability reduction, profit, food security, transformation, new knowledge, new technology, and productivity. By linking the above keywords together, we define climate-smart agriculture as a “transformative and sustainable kind of agriculture that tries to increase efficiency (productivity) in food security and production systems, using a combination of the pillars of climate change (adaptation, resilience, and mitigation) as well as smart and new technological knowledge, that do not only build capacity of farmers’ in terms of farming techniques, but also increase profit, reduces vulnerability of the systems as well as their results (farm products/animals), through the reduction of GHG emissions.” 

**Figure 1:**
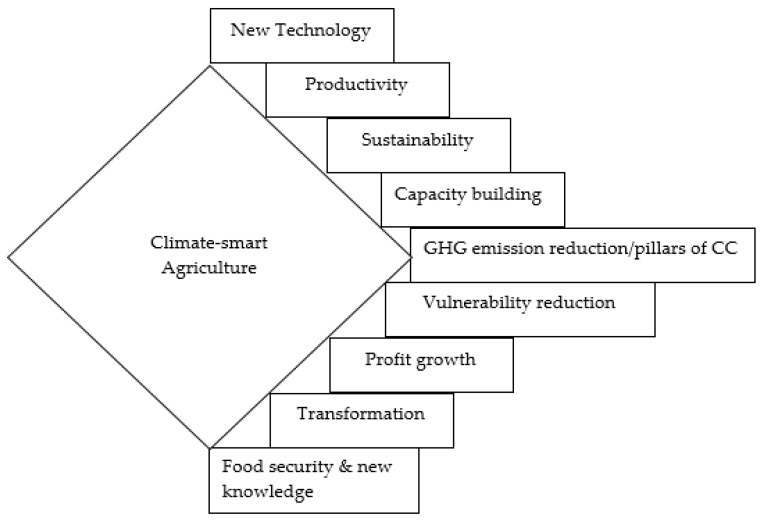
Key aspects of climate-smart agriculture (CSA).

While it can be argued that the list of keywords suggested within the current study is not exhaustive, many other definitions tend to be built around at least one of these keywords. Figure 1 is a diagrammatical representation of the main aspects of climate-smart agriculture for which it stands as a significant part of a smart village. The implication of the above expository listing of the fundamental parts of climate-smart agriculture means that for a smart village to be so called, it must strive to maintain within its agricultural systems all different aspects of CSA. Furthermore, other aspects of the smart culture within the smart village setting; smart energy management, smart living and smart healthcare, etc., must tap from these fundamental attributes of CSA, in order to provide robust services in their smart village functions. 

In demonstrating whether CSA could increase rice yield in China, Xiong et al. [79] used crop simulation models; version 0810 of the Environmental Policy Integrated Climate (EPIC) model [80], and version 4.0 of the so-called DSSAT, an acronym for Decision Support System for Agro-technology Transfer [81], respectively. It was observed that these software simulations that gave ideas on cultivar improvement and optimization of management practices for rice due to climate change, led to increased rice production. The EPIC models specifically yielded over 2000 kgha^−1^ during the 30-year period under review [79]. 

Rural African farmers tend to suffer a lot from adverse weather conditions. This further creates a need for cheap and reliable weather forecast system. To attend to such needs in Nigeria [82], a cheap automatic weather station that functions on solar energy was designed. By linking meteorological sensors to microcontrollers, the farmer could gain access to processed information related to weather, through a television screen. A thermometer collects temperature information, while the anemometer and LDR measures wind speed and sunlight, respectively. Embedded temperature sensors within the microcontroller receives analog information gathered by the thermometer and converts it to digital signals [82]. In some cases, unprocessed data can also be sent to farmer’s mobile phones. The cheap rate of the unit shows that it can serve as a very good system for crop management and food security, in the least developed nations. 

In a research carried out by Tenzin et al. [83], to ensure effective weather monitoring around a farm, the authors designed a very cheap cloud-based weather measurement unit, using an integration of different unique weather sensors. The system, which is made up of a base and a weather station, as well as a display unit, is capable of effectively gathering humidity, temperature, wind direction, wind speed, and many other weather data types. By experimenting its usage and statistically analyzing gathered data, it was observed that the unit provided similar results as the Davis Vantage Pro2 weather monitor, which was pre-installed on the same farm, thus, offering a cheaper option [83].

In a bid to design an integrated farm that efficiently manages water and reduces climate-demanding inputs, Doyle et al. [84] designed an aquaponics unit for vegetables and fish. The design consists of a 12V DC pump that delivers water from the fish tank to the flood tank, which then supplies the area where the crops are planted at a constant rate. As soon as water is removed from the fish pond, it is carried by gravity through the grow bed area, where it is stored until it is needed for watering the vegetable bed. The pump is powered using a solar panel of 150-Watt with a 120 Ah battery.

Having described some smart agricultural and climate-smart agricultural studies, it is important to note that while smart agriculture is mostly developed, research on CSA is relatively new and still at the level of policy and framework description [85]. In a systematic review study by Chandra et al. [85], the authors observed that research on CSA is mainly divided into three parts; global policy and plans around the world concerning further development of the concept, scientific research directions, and integration of pillars of the concept (which includes; adaptation, resilience, mitigation, and food security). With respect to CSA policy framework developed by the World Bank, Taylor [86] faulted the fundamental make-up of the concept on the following grounds.

▪ There are no explicit conditions that can be referred to as success of CSA, which makes certain fundamental aspects like productivity, completely implicit.
▪ Being an important part of sustainability, resilience as pointed out within World Bank’s CSA framework is not defined, thus, leaving the term implicit.
▪ Given an absence of conceptual framework for CSA, literature relating to the topic are merely based on success stories of some normative research on agricultural improvement.
▪ CSA tries not to be involved with how consumer sovereignty influences food production around the world, towards the consumption demands of the elite.

Given these fundamental shortcomings of CSA [86] ‘climate-wise food system’ is suggested as a more direct term that should be used to refer to sustainable food production systems, rather than CSA. Another criticism on the policy and framework of CSA comes with the injustice meted to smallholder farmers, as a result of the implementation of the concept [87]. By administering interview to some CSA experts, analysis based on a number of ethical positions showed that implementation of climate-smart agricultural approaches is not fair, especially with respect to allocation of income benefits and challenges of cost associated with emission reduction [87], among smallholders farmers and small agricultural processing industries. Budiman [87] further argued that based on how climate justice works, sharing of income benefits should depend on the financial capability of farmers.

In a comparative study of Philippines and Timor-Leste, five important features of climate-smart agricultural practices were observed by Chandra and McNamara [88]; strategies at country-specific institutional levels; delegated financial procedures; the state of the market; technology; and knowledge. In the two countries, CSA was used to resolve climate vulnerability challenges more than it was associated with emission reduction goals [88]. Overall, the researchers observed that advancing the course of CSA in these countries might involve multi-stakeholder approaches that cuts across different levels of participation, both within and outside the farm, rather than mere technical CSA developmental inputs [88]. From the above arguments for and against CSA, it is clear that while there are still fundamental challenges revolving round the CSA concept, the terms might likely continue to be utilized for agricultural problem solving, until it attains uniformity and intersection of ideologies, amongst researchers and policy makers. 

### What does Smart- and CSA Offer Smart Villages?

Having described in previous sections how the concept of CSA has evolved amidst the challenges faced within its developmental framework, an examination of the utility of climate- and technology-driven agriculture to smart villages is important. According to Azevedo [5], there is a big chance that CSA will empower and strengthen the conceptualization and execution of smart village in different ways. Safdar and Heap [89] noted that development of small grids to power certain climate-smart technologies has so far spurred a re-imagination of the possibility of home solar powering in many Indian villages. Items such as solar lanterns, and street solar lighting systems have become very popular. Nevertheless, a new concern is the way to enhance local productions and repairs of these materials, in order to cater for higher tariffs of importing them to interior villages, and shipping them back for repairs, when the items develop technical faults. The report also stressed how CSA has so far upheld gender equality, for instance, the CCAFS project in Kenya’s Nyando valley has mostly favored women whose incomes have improved due to new technology for growing their vegetables [13].

In documenting how CSA could provide smart village farmers with possible economic benefits, Khatri-Chhetri et al. [90] carried out a research using farmers of India’s Indo-Gangetic Plains. Major CSA practices by the farmers include diversifying crops, land levelling using laser, nutrient management in a site-specific mannerism, management of residue, and zero tillage, among others. The researchers started by calculating how much the farmers spent to adopt three most prominent CSA systems (variety of crops, land levelling using laser, and zero tillage). These values were estimated as +1402, +3037 and –1577 INR ha-1, respectively, for rice-wheat cultivation system. By improving their varieties in terms of crop production, the study results showed that the farmers of the Indo-Gangetic Plains can have their net return increase to up to INR 15,712 per-hectare, per-year. Similarly, when cultivating wheat and rice with no tillage, farmers could make up to INR 6951 per-hectare, per-year, and INR 8119 per-hectare, per-year with laser-based land levelling. Given the analyses of this results, it implies that integrating individual systems together would result in an even higher yield as well as income for the farmers. In econometric terms, adoption and execution of CSA practices for crop production in the north Indian River plain would significantly influence the cost of production, which decreased, but produced an increased yield of rice and wheat.

Scherr et al. [78] reported that CSA offers to rebrand villages by providing them with embrace ‘climate-smart landscapes’. This means that integrated landscape management principles that adopts the pillars of climate change must be in place prior to agricultural land allocation. The development of CSA objectives also requires strong institutional mechanism. When such systems are in place, its effects transcends to other parts of the village. Steenwerth [74] noted that while smart village residents might consider migrating to big cities, climate-smart agriculture could cause a rethink, as it gives room for entrepreneurial development in the agricultural sector, as seen in the case of youth training embarked upon in rural areas across Europe. Additionally, CSA also caters for increased demand for food due to the world’s growing population. This is achieved through methods that do not jeopardize environmental health [74]. With respect to animal husbandry, some zoonotic diseases can be detected early, so that treatment plans are set underway to prevent the farmer from infection. CSA also motivates the achievement of sustainable development goals through agricultural practices that use techniques that can drive food security, improve resilience, and effectively manage emissions [70]. CSA practices are also able to curb environmental challenges related to water pollution through the use of agrochemicals [91]. A notable aspect where smart agriculture surpasses expectations is the possibility of using it as a tool for enterprise resource planning, through which the safety of agricultural products/foods can be monitored [46].

## 4. Discussion

### Revisiting the Research Question

How will smart- and climate-smart agricultural research give account of, and conceptualize transformation and change in the smart village context?

In responding to the modified research question above, it is important to draw important ideas from the definitions of smart- and CSA. Albeit, CSA bears all characteristics of smart agriculture, with a step further in lowering GHG emissions. Consequently, accounts of conceptualizing transformation and change in smart village context might tend towards the adoption of key aspects of climate-smart agriculture (see Figure 1), which are somewhat multi-disciplinary in nature [92]. What this implies is that for smart villages to reach desired level in terms of development through research and policy frameworks, ideas of climate-smartness must be fully embedded across the facets of smart village agenda. According to Katara et al. [93], continuous adoption of new technologies is the first way to conceptualize transformation of smart villages. Since technology is bound to continually change, it becomes easy to bring evolving and smarter changes to smart village progress. This means that rural population must fully embrace ICT, especially since smart village idea is based on the fact that technology is adopted to hasten the growth of sustainable development [93]. Secondly, efficiency and productivity are not completely new words in smart village research. Nevertheless, it might be useful for smart village policy analysts to learn from prevention of losses for which CSA is known [94]. Another important aspect through which transformation of smart villages can be conceptualized is through capacity building of rural dwellers. As in the case of climate-smart agriculture, building capacities would bring about self-sufficiency for persons within these communities, thus reducing urban migration [15,19]. This is part of the current efforts within the different smart village initiatives. Of all the initiatives that smart village research can draw from climate-smart agricultural practices, the idea of seeking and promoting “new knowledge” [95] might be technically referred to as the most significant. Given that the world has now embraced a knowledge-based economy for which smart village development has to be a part. 

On the basis of towing a part of steady development in its processes, future smart village developmental projects need to adopt successful projects of the past as a yardstick for planning. For instance, tremendous success was recorded by the IEEE smart village initiative; the EU smart village-drive, as well as the CCACFS projects, to mention a few. By adopting the recipe for success within these projects, more smart-village projects would be actualized in many parts of the world. Furthermore, it is noteworthy to state that existing smart village projects also have unique challenges. Notable amongst the challenges faced by smart villages within the IEEE project is the issue of maintenance and repairs [96]. Although as part of the project framework, two individuals are often selected and trained within the villages to fix damaged smart inputs, when demands for these inputs become high, the number of technicians might no longer be sufficient to cater for repair and maintenance needs. This is one aspect where smart village development must learn from climate-smart ideas where capacity development is well-planned and readily available.

Another aspect for which smart village development can gain from climate-smart agriculture is in its sustainability approach. While CSA strives for the cheapest routes to progress in agriculture, smart village development mostly depends on donations and funding, which slows down the pace of making progress and achieving sustained growth. As a result, for any smart villages project to achieve lasting success, such a project would have to plan self-funding strategies [10], where inputs within the village is used to generate income that would fund new projects for growth, rather than unduly wait for funding before progress is made. In building its growth, smart village planners might need to prioritize new knowledge and link it to new technology for early warning measures against potential environmental disasters. Furthermore, proponents also need to ensure that pillars of climate change are largely considered in building infrastructures [13]. This is because the impact of climate change might continue to be felt for a long time. 

While ideas drawn from smart and climate-smart agriculture might indeed be useful for smart village development, Hargreaves et al. [97] explained that specific policies grounded in the values of rural areas are needed to help them transform into smart villages. This transformation must, therefore, bring effective utilization and management of resources within smart villages. The idea of transformation within the context of smart villages mostly draws attention to digital transformation, which is very important [98]. Another result of technological change is the social changes it brings [99,100].

Given the forgone discussion on how smart village development can be spurred from ideas borrowed from smart- and climate-smart agriculture, we argued that the development of a smart village has to be a gradual process. This is because the development must systematically and strategically prioritize the most important aspects, such as clean energy management and agriculture, bearing in mind the sustainability of the process.

## 5. Current Lessons & Future Research Direction

Overall, this study revealed a number of lessons from smart-agriculture and climate-smart agriculture ideas, which, if adopted in smart villages, would achieve the following goals.

▪ Improvement and optimization of existing smart village projects/processes in terms of precision and speed.
▪ Increased efficiency and productivity, which can lead to increased income/profit on ventures embarked upon by smart-village dwellers.
▪ Better planning brought about by efficient forecasting and prediction systems, which help to guide against potential dangers, and to take proactive steps in planning and preparation for such eventualities.
▪ Offer of cheaper and equally effective data gathering avenues for easy detection of challenges and problems.
▪ Reduced dependence on external funding, and a drive for self-sufficiency encouraged by innovation.

While the above lessons are specific to smart village development, there are specific shortcomings of climate-smart agriculture that must be noted [91,92], and which ought not to be adopted in smart village development.

1. Sain et al. [101] used cost-benefit analysis to analyze variability and uncertainty of some CSA parameters. It was observed that while CSA is generally promising, not all CSA parameters were indeed profitable in the long run [101].
2. With CSA comes IoT, Blockchain, and artificial intelligence in agricultural operations. As such, there is the challenge of helping rural farmers understand the operation of smart farm inputs, and interpretation of data gathered from the farms using CSA tools [102]. The situation might be worse in rural Africa, where farmers rarely have any level of formal education.
3. Interoperability is another serious challenge for adopting CSA. An example is described by Kalatzis et al. [103] in the use of gaiasense ^TM^ farming solution.
4. The cost of acquiring smart farming implements cannot be overlooked when listing some known challenges of CSA [104]. Smart sensors for instance are generally expensive [105]

As a result of some of the aforementioned challenges of CSA, future studies might look at the challenges posed by the adoption of “climate-smart” agriculture, prior to the full adoption of its fundamental aspects, as described in this study. This is because research by Taylor [86] pointed out certain foundation faults in the description of CSA by the World Bank group. There is also a fundamental problem in how CSA handles climate justice [87]. Another aspect opened to future research is the development of the climate-smart villages, as used in some studies [69]. While this has been achieved in some parts of the world today [13], it might be the case that smart-village research is yet to reach a maturity level as to warrant even more terms to be coined from it. 

## 6. Conclusions

The uniqueness of smart village projects around the world means that approaches towards smart village development might also differ. This study showed that smart and CSA are key areas that must be considered in developing a smart village project, and offer several lessons to proponents of smart village ideas, given how these concepts have enjoyed steady conceptualization in the research literature. Another important consideration that must be carefully explored is the tendency of developing smart villages in line with the concepts upon which smart cities are built. Having clarified in Section 1 that smart villages are not extensions of smart cities [106], it is important to understand that the challenges of rural areas differ significantly from those of cities. Hence, smart village development must come with uniquely defined plans and strategies for its development [107].

A major driving force for “smarting up” rural areas is the mass exodus of persons to the cities, as well as inferior services offered in these villages [107]. Nevertheless, an introduction of the smart village concept comes with new opportunities brought about by technology, which is currently touted as the major economic driver of the 21st century. The current study, therefore, tries to adopt the technological ideas of CSA in creating a foundational path for smart village development. To do this, the study carefully analyzes the framework of CSA and proposes that the same be adopted for developing smart villages. It is observed that certain fundamental aspects of technological innovation; productivity, new knowledge, new technology, capacity building, vulnerability reduction, increased profits, etc., are fundamental to the building of smart villages. Nevertheless, these fundamental terms cannot be embedded immediately. Rather, it must follow gradual process that gives priority to the important aspects.

## Figures and Tables

**Table 1:** Summary of some biological sensing techniques for soils and plants (Adapted from Antonacci [6]).

Group	Analyte	Bio-Sensing Method	Conversion	Nanomaterial Media	Detection Limit/Time	Reference
Herbicide	Soil glyphosate; soil glufosinate	Specified dual polymers with imprinted template	Anodic stripping voltammetry done with differential pulse that makes use of nanoparticles of gold adjusted pencil graphite electrode	Nanotubes with multi-walled carbon	0.35 ng mL^−1^; 0.19 ng mL^−1^	[30]
	Soil atrazine	Tyrosinase inhibition	Utilizing an amperometric analysis adopts a conventional 3 electrode cell	Nanotubes of titanium dioxide	0.1 ppt (approx. 600 s)	[24]
Fungus/Fungicide	Trichoderma harzianum present within the soil	DNA probe in a single strand	Electrochemical analysis that utilizes an electrode made from gold	Nanoparticles of zinc oxide- chitosan nanocomposite membrane	1 × 10^−19^ mol/L (600 s)	[31]
Fertilizer & Nutrient	Soil nitrates	Polypyrrole electrode that is in solid state, and easily selects ions	Experimenting a potentiometric analysis through the use of adjusted glass carbon	Oxide of graphene	0.00001 M (≤15 s)	[32]
	Soil nitrates	Reduction of nitrate	Carrying out an impedimetric analysis via the use of a gold electrode	Nano-fibers of poly(3,4-ethylenedioxythiophene) polystyrene sulfonate - nanosheets composite derived from graphene oxide	0.68 mg/L (few hundreds of seconds)	[29]
	Soil urease; Soil urea	Nanoparticles of gold is adopted as catalyst, acting like horseradish peroxidase	pH indicator; Colorimetric;	Nanoparticles of gold	1.8 U/L (600 s); 5 µM (600 s)	[33]
Disease	Ganoderma boninense (synthetic DNA)	DNA probe	Transfer of energy through fluorescence resonance	Quantum dots	3.55 × 10^−9^ M (600 s)	[28]
	Sweet corn seed: Pantoea stewartii sbusp. Stewartii NCPPB 449	Immuno-sensor	Immunosorbent assay linked to enzyme	Nanoparticles of gold	7.8 × 10^3^ cfu/mL (below 1800 s)	[34]
Virus	For orchid plant: Odontogloss um ringspot virus; Cymbidium mosaic virus;	plasmon resonance of particle; Fiber optic	Utilizing nano-rods made of gold as sensing device (Immuno-sensor)	Nano-rods made of gold	42 pg/mL (600 s) 48 pg/mL (600 s)	[35]
Pesticide	Soil acetamiprid	Affinity with 20mer specific aptamer	Carrying out an colorimetric analysis	Nanoparticles of gold	5 nM (300 s)	[26]
	Soil methyl parathion	Acetylcholinesterase inhibition	Adopting adjusted glassy electrode of carbon to cause voltametric differential pulse	Nanotubes with multi-walled carbon -chitosan nanocomposites	7.5 × 10^-13^ M (2 s)	[25]

**Table 2:** Definitions of CSA.

Definition	Keywords	Reference
The combination of activities that helps to: build adaptive measures that increase productivity, increase resilience to stresses posed by climatic change, and reduce GHG emissions.	Capacity building; emission reduction	[70]
A sustainable method through which improved productivity and income is achieved in agricultural production via the adoption of adaptation, resilience and GHG emissions mitigation	Sustainability; Emission reduction; productivity; profit; capacity building	[71]
Processes that transform agricultural systems to boost food security, given current changes in climate	Productivity; transformation; food security	[68]
A system of agriculture that supports emission reduction while creating improved productivity profits, nonetheless reducing vulnerability	Vulnerability reduction; emission reduction; profit growth	[72]
A system of agriculture that improves production in a sustainable manner, while building capacity to ward-off agricultural and climate change challenges	Sustainability; capacity building; productivity	[73]
Strategies that are able to curb agricultural challenges through the increment of resilience activities to extreme weather conditions, building adaptive capacities to climate change and mitigating agriculture-based GHG emission increase.	Capacity building; emission reduction.	[74]
Practices that add to improved food security globally, and further enable farmers to effectively adapt to the incidence of climate change and global emission levels	Capacity building; emission reduction; food security	[75]
Combined use of ultramodern technologies and processes that work together to boost farming productivity and incomes, while increasing the farm’s and farmers’ ability to manage climate change through GHG emission reduction.	New technology adoption; productivity, profit; capacity development; emission reduction	[76]
A technique that combines a number of sustainable techniques to fight particular climate challenges within a specified farming area	Sustainability; GHG emission reduction	[77]
An agricultural framework that tries to develop and adopt technique that will improve rural livelihoods, food security, and facilitate adaptation to climate change, while also providing mitigation benefits	New knowledge; food security; capacity building.	[78]
